# Process evaluation of health fairs promoting cancer screenings

**DOI:** 10.1186/s12885-017-3867-3

**Published:** 2017-12-18

**Authors:** Cam Escoffery, Shuting Liang, Kirsten Rodgers, Regine Haardoerfer, Grace Hennessy, Kendra Gilbertson, Natalia I. Heredia, Leticia A. Gatus, Maria E. Fernandez

**Affiliations:** 1 Rollins School of Public Health, Emory University, 1518 Clifton Road, NE, Atlanta, GA 30322 USA; 2 University of Texas School of Public Health at Houston, 7000 Fannin Street, Houston, TX 77030 USA

**Keywords:** health fair, early detection, cancer screening, health promotion, cancer education, ccommunity health education, breast neoplasms, cervical neoplasms, colorectal neoplasms

## Abstract

**Background:**

Low income and uninsured individuals often have lower adherence to cancer screening for breast, cervical and colorectal cancer. Health fairs are a common community outreach strategy used to provide cancer-related health education and services.

**Methods:**

This study was a process evaluation of seven health fairs focused on cancer screening across the U.S. We conducted key-informant interviews with the fair coordinator and conducted baseline and follow-up surveys with fair participants to describe characteristics of participants as well as their experiences. We collected baseline data with participants at the health fairs and telephone follow-up surveys 6 months following the fair.

**Results:**

Attendance across the seven health fairs ranged from 41 to 212 participants. Most fairs provided group or individual education, print materials and cancer screening during the event. Overall, participants rated health fairs as very good and participants reported that the staff was knowledgeable and that they liked the materials distributed. After the fairs, about 60% of participants, who were reached at follow-up, had read the materials provided and had conversations with others about cancer screening, and 41% talked to their doctors about screening. Based on findings from evaluation including participant data and coordinator interviews, we describe 6 areas in planning for health fairs that may increase their effectiveness. These include: 1) use of a theoretical framework for health promotion to guide educational content and activities provided, 2) considering the community characteristics, 3) choosing a relevant setting, 4) promotion of the event, 5) considerations of the types of services to deliver, and 6) evaluation of the health fair.

**Conclusions:**

The events reported varied in reach and the participants represented diverse races and lower income populations overall. Most health fairs offered education, print materials and onsite cancer screening. Participants reported general satisfaction with these events and were motivated through their participation to read educational materials or discuss screening with providers. Public health professionals can benefit from this process evaluation and recommendations for designing and evaluating health fairs.

## Background

Breast, cervical and colorectal cancer are three frequently diagnosed cancers with known prevention and early detection strategies. Breast cancer is the most frequently diagnosed cancer and second cause of cancer death in women [1]. Additionally, colorectal cancer is the second-most common cancer diagnosed and the second leading cause of cancer mortality among cancers affecting both men and women [1]. Only 59% of adults were up-to-date with colorectal cancer screening in the U.S. [2] Cancer screening can reduce the incidence and mortality of these cancers [1, 3]. Screenings for both breast and cervical cancer have not increased from 2008 to 2010 and meeting of the Healthy People 2020 targets for cervical and breast cancer screening may be challenging [4].

The Community Guide to Preventive Services, which offers evidence-based recommended interventions for public health based on summarized findings from systematic reviews, has recommended strategies focusing on patients, providers, and health systems to increase breast, cervical, and colorectal cancer screening [5]. A recent systematic review and qualitative study of coordinators of special events, such as health fairs, that focused on cancer screening found that these events often combined one on one or group education, small media (e.g., print materials) and reducing structural barriers (e.g., transportation, onsite screening). These are all Community Guide recommended strategies [6, 7]. Health fairs are community events that offer education about health topics typically through educational booths and materials [8–10]. They also offer health screenings or referrals, and offer medical and community outreach training for healthcare professionals (e.g., nurses) [11–13].

Evaluations of health fairs for promoting cancer screening are limited [12] and most data are collected at or immediately following the event. Evaluation measures for these events often focused on screenings delivered at the event [13–15], ratings of logistics [16], and satisfaction [13]. Longer term follow-up is necessary to document health fairs’ meeting of their goals, implementation and impact of these events, such as discussions with providers about screening or completion of screenings. Therefore, evaluation of health fairs is needed to learn more about their goals, implementation and outcomes. These data will contribute to the literature on community-based interventions to promote cancer screening.

The purpose of this paper is to describe the results of the process evaluation of 7 health fairs. This evaluation will help answer these questions about reach, services delivered, perceptions of the event and effects of the fairs. We sought to answer the following research questions: 1) What was the reach of the health fairs? 2) What were common strategies employed across the fairs? and 3) What were the levels of participant satisfaction and perceived impact of the fairs? Based on the analysis of these data, we provide recommendations for planning, implementation, and evaluation of health fairs.

## Methods

We conducted a process evaluation of seven health fairs using a mixed-method approach that included administration of baseline surveys during the fairs and a 6-month follow-up by phone. We also conducted in-depth qualitative interviews with fair coordinators, and reviewed administrative records of attendance and services delivered at the fair. The evaluation protocol for the study was approved by the Emory University and the University of Texas Health Science Center at Houston Institutional Review Boards prior to beginning recruitment.

We identified 2 community partners that were offering health fairs through the University of Texas (subcontractor to the Emory grant) and then developed a request for application for other sites to participate in the evaluation of a health fair. The announcement was sent to grantees of different cancer programs that received funding from the U.S. Centers for Disease Control and Prevention (CDC; e.g., community based and tribal organizations, health departments, cancer coalitions) and community partners of a national Cancer Prevention and Control Research network (8 universities). A review panel of eight health educators and prevention scientists selected five sites from 30 applicants to participate in the evaluation project. Selected sites received $5000 for participating in the evaluation and/or financial assistance to provide resources at the event. The study team developed data collection protocols and measures to be used across health fairs.

Two study staff attended the health fairs and were located in the fair registration area. They invited adults to participate in the evaluation at the time of fair registration. Participants who satisfied the following criteria were recruited: 1) aged 18 or older, 2) spoke English or Spanish, and 3) attended the health fair. Participants complete a written informed consent form and a self-administered baseline survey before they left the registration area and attended the fair events. Participants provided information about knowledge about breast, cervical and/or colorectal cancer, factors related to cancer screening, and screening behavior at the fairs. After 6-months, participants were contacted by telephone to complete a follow-up survey to assess similar items and process evaluation questions about the health fair. We conducted baseline surveys at the health fairs from May to September 2013 and conducted 6-month follow-up surveys through June 2014. To increase the response rate at follow-up, we asked for alternate contacts and contact information from the participants and tried to contact them up to 7 times. Participants received a $15 gift card for their participation for each of the two surveys. Within a month after each health fair, the health fair coordinator was interviewed by research staff about the purpose of the fair, key delivery strategies, barriers to implementation and feedback on the fair.

The baseline survey included questions on demographics, including gender, age, race, education, employment, income level, and insurance status. Questions on knowledge about breast, cervical and/or colorectal cancer, facilitators and barriers to screening, and screening history (prior to the health fair) were also included, but are not the focus of this process evaluation. The 6-month follow-up survey included the same items at baseline, including cancer screenings received since the health fair, as well as process evaluation questions about the health fair. We attempted to conduct the process evaluation on a half of the baseline participants due to response burden and staff constraints. There was a random selection of 50% of baseline participants per site. These participants were comprised of both screening-eligible participants and non-screening eligible participants to get a breadth of responses. Non-screening eligible participants, or those who were adherent to screening, were asked to complete the process questions and were not asked about cancer screening.

For this paper, we are focusing only on demographics collected at baseline, process evaluation questions from the follow-up surveys, and each health fair administrative data. The process evaluation items included an overall rating of the health fair on a scale of 1 = poor to 4 = excellent and ratings of importance of the fair in getting screening and of health fair elements (e.g., location, staff, logistics) on a scale of 1 = strongly disagree to 5 = strongly agree. In addition, we assessed participants’ receipt of information at the fair and behaviors related to screening information seeking (i.e., reading more about cancer) following the health fair. Several open-ended questions allowed participants to describe aspects of the health fair they liked and disliked and suggestions for improvements. The key informant interviews with health fair coordinators included 72 questions and covered: 1) general health fair information; 2) goals, recruitment and participant demographics; 3) activities and delivery (i.e., services offered, use of theory in planning health fair activities and health education materials); 4) fair results (screening outcomes, process data); 5) benefits, barriers, recommendations; and 6) demographics of the coordinator [7]. Additionally, coordinators completed a standardized cost form documenting expenditures for the fair.

We analyzed and presented data for each of the seven individual health fair sites separately and for the total participants across fairs. We used SPSS version 22.0 for all analyses [17]. We ran descriptive statistics for all demographic and process variables. Data about the health fair from the interviews and administrative records included location, date, cancer focus, population of interest and expected/actual reach, services offered onsite, provision of onsite screening, and costs were abstracted and presented in a table. The services offered were categorized into effective strategies to promote cancer screening recommended by the Community Guide to Preventive Services Taskforce and these were also noted in the summary table. For the open-ended items from the survey, research staff read each question and categorized the responses into general themes for what the participants liked most and least and recommendations for improvements. For the key informant interviews, research staff abstracted key program components and lessons learned from the notes. We triangulated the data during three meetings through a discussion of key health fair domains (e.g., promotion, delivery, etc.), a review of the process evaluation ratings from 6-month follow-up and the key informant interview notes. The research team then made the recommendations for key guidance for the design of health fairs for future planners.

## Results

Table 1 provides descriptions of the health fairs. All seven health fairs addressed breast cancer; six of them also addressed cervical cancer; three of them addressed breast, cervical and colorectal cancer, and two provided information on skin cancer and prostate cancer. The organizations hosting the health fair varied from community based organizations to local health departments. Most had reported previous experience with health fairs. The target populations of these health fairs were mostly low-income, underserved, minority, uninsured or underinsured. There were 589 baseline surveys completed at the fairs. All health fairs provided informational materials such as brochures, handouts, and fact-sheets from different sources including Susan G. Komen, American Cancer Society and the CDC. They also provided education on cancer screenings conducted by health professionals: two invited physicians to give presentations about cancer screening (group education), one had clinicians conducting individual health counseling, and two had health educators or promotoras (community health workers) teach women how to conduct breast self-exams. All seven health fairs provided onsite screening and three of them also provided referrals to increase access to screening services among underserved populations. All but two fairs provided clinical breast exams onsite; three offered mammograms onsite for no or very low cost; two each provided pap smears or FOBT/FIT tests; one gave out vouchers for breast and cervical cancer screening at designated providers. Completed onsite cancer screening ranged from 2 clinical breast exams to 44 Pap tests. Furthermore, two fairs also offered assistance to participants in applying for state cancer screening programs and make appointments for cancer screening. The total costs (including in-kind contributions) of these health fairs ranged from $6586 to $43,428.

**Table 1:** Health Fair Descriptions, Screening and Costs

Site Number, Location (City, state)	Date(s)	Cancer of focus	Urban/Rural	Target audience (description and estimated number of participants)	Key Fair Components	Onsite Screening/Referrals	Total Cost (including in-kind contribution)
Site 1 Church Atlanta, GA	7/20/2013	Breast & Cervical Cancer	Urban	Church members in the Old Fourth Ward and residents aged 40 and older who are predominantly African American and female. (*N* = 212)	Cancer-Related Activities: • Guest speakers provided “Ask the Doctor” session specific to women’s cancer. Speakers included HPV expert, OBGYN (youth reproductive health specialist), OBGYN oncologist, health professionals from ACS (client navigator) • Provision of patient resources: navigators from Grady Hospital, director of the American cancer society who brought ACS brochures, information and resources • Center for Black Women’s Wellness gave two sessions (information on breast and cervical cancer screenings) and provided information for future screenings Screening: • Offered onsite mammograms Community Guide Strategies: Group Education, Small Media, Reducing Structural Barriers	• Onsite mobile van provided mammograms	$43,428
Site 2 Community clinic Billings, MT	9/28/2013	Breast, Cervical & Colorectal Cancer	Rural	Low-income, underserved, minority, migrant, and underinsured/ uninsured populations in Yellowstone County and surrounding communities. (*N* = 200)	Cancer-Related Activities: • Helped women in need apply for the Montana cancer screening program • Provided Information on tobacco prevention, nutrition program, colorectal cancer screening and breast self-examination Screening: • Provided pap smears, cervical exams, clinical breast exams, mammograms and colon care at-home screening tests • Provided skin checks – and covered health topics on skin cancer Other: • Provided info on housing programs – which provides housing options for cancer patients in need • Provided info on local Women, Infants & Children (WIC) and other programs Community Guide Strategies: Small Media, Reducing Structural Barriers	• Mammograms [14] • Pap smears [8] • Provision of colorectal stool guaiac card (50)	$24,417
Site 3 Local health department Bronson, FL	9/28/2013	Breast, Cervical & Colorectal Cancer	Rural	Breast Screening - women over 40; Cervical - women 21 and older; and CRC - Men and Women over age 50. (*N* = 63)	Cancer-Related Activities: • Had booths with education on breast and cervical cancer. One booth had a breast model with lumps. An educator instructed women on the proper way to identify lumps and educate the women on proper breast self-exams. Susan G. Komen provided educational materials. • Offered the HPV shot and had STD information from CDC. • Colorectal- booth contained men’s health information. A male nurse offered one-on-one education for men Screening: • Florida Department of Health (FDOH) offered an early detection program- offered the opportunity to sign up to get Pap smear at participant’s convenience. Provided educational info-graphics. Community Guide Strategies: Small Media, One-on-one Education, Reducing Structural Barriers	• Clinical breast exams [5] • Pap smears [14] • Provision of FOBT test kits [17]	$16,123
Site 4 State health department Farmington, ME	9/16–17/ 2013	Breast, Cervical & Colorectal Cancer	Rural	All ages, with the majority of participants from a rural or remote County, and meet the definition of being a disparate population. Targeting adults and lower SES. (*N* = 200)	Cancer-Related Activities: • Provided information on: - Ways to prevent colon cancer - Breast care brochures - Prostate brochures • Distributed brochures about skin cancer and smoking and smoke-less tobacco products and info about Maine tobacco help line Screening: • Provided information about Maine breast and cervical health program – especially for those who may not have insurance; help the eligible make appointments on the spot • Provided info on Screen Me for Life initiative through DHHS and CDC for those due for colon cancer screening, info geared for men and women Other: • Health counseling provided by health educators, RNs, Nurse practitioners, medical doctors Community Guide Strategies: Small Media, Reducing Structural Barriers	Clinical breast exams [2]	$8525
Site 5 Tribal community based org. Valley Center, CA	7/12/2013	Breast	Rural	American Indian/Alaskan Native women 42 years and older and non-NA women eligible for the California Cancer Detection Program (CCDP) (*N* = 100)	Cancer-Related Activities: • Invited speaker (Physician of the organization) presented about breast cancer and reasons why women should have a mammogram and clinical breast exam • Offered group education on breast health and provided handouts and brochures • Provided prizes specific to breast cancer (i.e. diaries to track when you had a mammogram, tote bag) Screening: • During screening, each lady was asked when they had their last exam and they will be followed-up for their next mammogram screening Community Guide Strategies: Small Media, Group Education, Reducing Structural Barriers	Mammograms [18]	$6586
Site 6 Community based org. Houston, TX	5/19/2013	Breast and Cervical Cancer	Urban	Hispanic women (*N* = 68)	Dia Del La Mujer Cancer-Related Activities: • Partnered with local Spanish station, Univision TV to promote the event • Breast and cervical cancer education given by 22 promotoras; usage of breast model to teach self-breast exam by onsite Patient Navigators Screening: • Clinical breast exams offered at health fair (Onsite Providers: Methodist Hospital and Baylor College of Medicine) Community Guide Strategies: One-on-one education, Group education, Small media, Client incentives, Reducing Structural Barriers, Reducing out of pocket costs	• Onsite clinical breast exams (68) • Vouchers for breast and cervical cancer screenings (68)	$12,550
Site 7 LGBT-serving Community based org. Houston, TX	9/7/2013	Breast and Cervical Cancer	Urban	Latina LGBT women and transgender men (*N* = 41)	Screening • Well person exams including clinical breast exam and Pap tests provided onsite (Memorial Hermann Neighborhood Center) • Breast exams provided through Breast Health Collaborative, The Rose and MD Anderson Cancer Center mobile unit • 3 patient/nurse navigators onsite who referred patients for breast/cervical cancer screening services Other: • Training providers on LGBT cultural for November Health Fair for breast cancer screenings • Community Guide Strategies: One-on-one education, Small media, Client reminders/calls, Client incentives, Reducing structural barriers, Reducing out of pocket costs	•Onsite clinical breast exam [22] •Cervical cancer screenings [22]	$14,479 & 321 volunteer hours

Note. Estimated number of participants were those who attend attended the fairs. In most cases, those who enrolled were almost the same as estimated attendees except for 1 site

### Results from process evaluation items

Table 2 describes the characteristics of participants (collected at baseline) who responded to the follow-up survey (*N* = 249). The participants were female (94%), 50 to 75 of age (49%), White (42%), had some college education (35%), employed (55%) and low income (49% with annual income < $25000). About 37% of them had no insurance. At baseline, screening adherence varied between cancers: 57.2% for colorectal, 73.5% for cervical cancer and 79.1% for breast cancer.

**Table 2:** Demographic Characteristics of Health Fair Participants (*N* = 249)

Characteristic	Number	Percent
Gender (*n* = 201)		
Male	12	6%
Female	189	94%
Age (years) (*n* = 184)		
18–29	16	9%
30–39	26	14%
40–49	46	25%
50–75	90	49%
75+	6	3%
Race (*n* = 217)		
White	91	42%
African American	77	35%
Asian	4	2%
Alaskan Native	30	14%
Other/Multi-race	15	3%
Ethnicity (*n* = 180)		
Hispanic/Latino origin	40	22%
Education (*n* = 226)		
Less than high school	21	9%
High school	48	21%
Some college or technical school	79	35%
College	52	23%
Post graduate degree	24	11%
Income (n = 212)		
Less than $10,000	54	25%
$10,001–$25,000	50	24%
$25,001–$50,000	54	25%
$50,001–$70,000	22	10%
$70,000 or more	32	15%
Employment Status (*n* = 225)		
Unemployed	64	28%
Employed	123	55%
Retire	38	17%
Insurance Status (*n* = 227)		
Insured	144	63.4%
Uninsured	78	34.3%
Don’t know	5	2.2%

Note. All variables have different N’s due to missing data.

The provision of the educational materials, tests, or testing appointments the participants received varied across health fairs. The majority of participants (approximately 85%) reported receiving educational and print materials; 13% received screening tests and referrals. Only 4% received assistance in making appointments for cancer screenings. Nineteen percent received additional unspecified materials or services.

On average, 80% of participants rated the health fairs as an important factor in their decision to get screened, ranging from 55% (Site 2) to 88% (Site 5). When asked about the importance of the health fair in their decision to get screened, some mentioned that it was a reminder of the importance of screening, and provided an opportunity for people without insurance to get screened (cited this as their main reason for attending the fair). Many people believed that the fairs raised community awareness about the importance of screening, and provided education to those who would not otherwise have gotten it.

Additionally, participants rated the health fairs on a variety of elements (Table 3) on a 4-point Likert scale (1 = poor to 4 = excellent). The majority of participants (60%) thought that the health fair was good or excellent (M = 3.59, SD = 0.56) with a range of 3.36 to 3.85. Over 90% of participants found the location convenient, the staff knowledgeable, the information useful and easy to understand, and the fair easy to navigate and well organized. Participants overall indicated that they were less satisfied with the communication that they had about the event (M = 3.74, SD = 1.27); however, they felt that the location was convenient (M = 4.36, SD = .81). Almost all (99.5%) thought that the respective organizations should hold similar events in the future.

**Table 3:** Rating of Health Fairs and Behaviors Performed after Fair (*N* = 249)

	Total (*n* = 249)	Site 1 (*n* = 43)	Site 2 (*n* = 11)	Site 3 (*n* = 42)	Site 4 (*n* = 99)	Site 5 (*n* = 16)	Site 6 (*n* = 26)	Site 7 (*n* = 12)
	Mean (SD)	Mean (SD)	Mean (SD)	Mean (SD)	Mean (SD)	Mean (SD)	Mean (SD)	Mean (SD)
Ratings of the Fair^a^								
Opinion of the health fair	3.59 (.56)	3.65 (.53)	3.36 (.67)	3.62 (.54)	3.47 (.61)	3.69 (.48)	3.85 (.37)	3.83 (.39)
Prior to the health fair, I received sufficient information about the fair	3.74 (1.27)	4.07 (1.22)	3.73 (1.01)	4.21 (.87)	3.14 (1.18)	4.06 (1.18)	4.00 (1.63)	4.83 (.39)
The fair was held in a convenient location	4.36 (.81)	4.28 (.77)	4.55 (.93)	4.45 (.67)	4.27 (.70)	4.50 (.73)	4.46 (1.30)	4.42 (1.00)
The staff were knowledgeable	4.54 (.67)	4.72 (.50)	4.36 (.81)	4.31 (.90)	4.41 (.62)	4.81 (.40)	4.88 (.59)	4.75 (.45)
The information provided was useful	4.54 (.60)	4.81 (.45)	4.27 (.65)	4.48 (.67)	4.29 (.61)	4.69 (.48)	5.00 (.00)	4.92 (.29)
The materials provided were easy to understand	4.48 (.66)	4.72 (.50)	4.36 (.81)	4.38 (.62)	4.26 (.65)	4.75 (.45)	4.81 (.80)	4.83 (.39)
I understood the information presented to me	4.49 (.58)	4.72 (.45)	4.45 (.52)	4.33 (.61)	4.25 (.58)	4.75 (.45)	4.96 (.20)	4.75 (.45)
It was easy to move around the fair	4.43 (.69)	4.37 (.76)	4.55 (.52)	4.48 (.77)	4.24 (.66)	4.63 (.50)	4.85 (.61)	4.83 (.39)
The health fair was well organized	4.50 (.63)	4.58 (.59)	4.45 (.93)	4.48 (.63)	4.29 (.59)	4.81 (.40)	4.92 (.27)	4.75 (.87)
Behaviors Taken after Fair, n (%)								
Read information about colorectal, breast, or cervical cancer	151 (60.6)	32 (74.4)	4 (36.4)	24 (57.1)	54 (54.5)	13 (81.3)	16 (61.5)	8 (66.7)
Talked to a doctor/health provider about colorectal, breast, or cervical screening	102 (41.0)	23 (53.5)	7 (63.6)	15 (35.7)	39 (39.4)	9 (56.3)	6 (23.1)	3 (25.0)
Had a doctor/provider talked with you about colorectal, breast, or cervical screening	106 (42.6)	20 (46.5)	6 (54.5)	17 (40.5)	38 (38.4)	11 (68.8)	9 (34.6)	5 (41.7)
Had a conservation about colorectal, breast or cervical cancer with family, friends or other people in your community	146 (58.6)	31 (72.1)	6 (54.5)	23 (54.8)	46 (46.5)	13 (81.3)	21 (80.8)	6 (50.0)

^a^Ratings were 1-Poor to 4 = Excellent. Remainder were 1 = Strongly disagree to 5 = Strongly agre

Table 3 summarizes participants’ self-reported behaviors related to screening information-seeking within six months after the fairs. On average, 60% of participants read additional information about the relevant cancer screening, with frequencies ranging from 36% to 81% across health fairs. About 41% reported speaking to a health care provider, and 43% reported that a health care provider spoke to them about cancer screening. Approximately 59% of participants said they had spoken with family, friends, or community members about breast, cervical or colorectal cancer since attending the event.

Participants also provided additional qualitative feedback about the health fairs on the survey. When asked what they liked most about the health fairs, participants noted that they enjoyed the variety of speakers, including health care providers, cancer survivors, and other panelists. Some participants reported that the “friendly environment” as a strength. They appreciated that health fair staff employed a “no-pressure” approach and that participants freely interacted with each other in encouraging ways. Participants also enjoyed the variety of recreational activities (e.g., basket weaving), and the opportunity to ask questions of health care providers. On the health services side, participants valued the onsite screenings available, and appreciated the assistance provided in making appointments on the spot. Participants also commented on the variety of health topics covered and the different vendors offering information and materials. Participants also liked the gift cards, free food, and other small gifts provided. Participants remarked that the written materials allowed them to take what they had learned home to family and friends who did not attend.

While many participants did not have any negative comments about the health fair, some criticisms that participants mentioned included the logistics, such as the fair venue’s inconvenient location, inadequate size, overcrowding, and the weather. Many people thought the fairs need more advanced advertising, including running ads on radio or television. Others thought the fairs would have been improved if there had been more attendees and a greater variety of vendors. They also suggested increasing publicity of fairs, specifically by doing more community outreach, increasing health topics covered, vendors and attendees, and the variety of speakers, as well as providing more educational materials. In terms of logistics, some suggested that fairs be held more often and at more convenient locations accessible by public transportation, have more space or an alternate venue in case of bad weather, had more staff, and are better organized. Lastly, some participants attending fairs that did not offer same day screening services stressed the importance of having more providers for screenings, offering screenings for free, and having screenings available at the fair.

### Results from health fair coordinator interviews

From the coordinators’ perspectives, all health fairs were successful, though at different degrees, with three of them being moderately successful and two being very or extremely successful. An important element of the perceived success was the completion of cancer screenings onsite for those who would not have gotten screened had they not attended the health fair. One site coordinator also noted the engagement of participants in their activities as an element of success. Besides providing education and screenings, most coordinators reported other benefits for the participants, organizations, communities as well as themselves. Some participants were offered free rides to and from the health fairs so they could stay for the entire fair and learn about other health topics. Participants also received small “prizes” (e.g., tote bags, diaries to track screening, etc.). Three coordinators suggested that the health fairs created community dialogues around cancer and other health issues, fostered a sense of community connection and cultivated positive changes in the community. The organizational benefits included networking with other organizations and community partners, and leveraging resources from other organizations (e.g., some labs provide support for expenses of genetic testing for cancer). Two coordinators mentioned that they have benefited personally from the fair by learning more about the populations that they serve, improving communication skills, as well as improving their grant writing skills.

Conversely, coordinators also noted that implementing health fairs was a major undertaking with some challenges. Planning for the fairs was time consuming and involved a great deal of administrative burden, including inviting speakers, scheduling events and activities with multiple vendors, as well as setting up booths, materials and screening equipment onsite. Working out the budgets and finances for the fairs was also a challenge with the majority of the funds, resources and services being in-kind donations. In addition, all health fairs involved volunteers and some had a large number of them (up to 85 for a single-day event). Therefore, the health fair planning required a lot of time and effort for training. Coordinators cited inconvenient locations, bad weather, lack of community interests, and an abundance of other health fairs in the area as barriers to participation.

The coordinators also offered advice for practitioners who are conducting fairs. The most common recommendation was to start planning earlier and to set aside more time for scheduling activities and training volunteers. To increase participation, coordinators suggested selecting a venue that is closer to a main road, involving the media (both mass and social media) sooner to increase awareness of the health fairs among the target populations, understanding participants’ needs and interests and providing incentives accordingly. For screening referrals, one coordinator suggested using client reminder cards with appointments.

We developed a guide based on the study findings to help practitioners determine what steps should be taken prior to, during, and following a health fair (Table 4). Key guidance for practitioners and decision makers who are planning to conduct health fairs include: 1) allocating sufficient amount of time for planning and training are key for implementation success; 2) involving mass, small and social media in the early stage of planning process to increase community awareness and solicit interest among target population to increase participation; and 3) building partnerships and fostering collaborations to leverage resources for these events. We have shared these recommendations with CDC cancer coalitions and programs and will continue to disseminate this guide through local and regional health promotion conferences.

**Table 4:** Key Considerations for Developing a Health Fair

Health Fair Element	Consideration
Theoretical Framework	• Consider social or behavioral theory in the design for the health promotion content and delivery • Develop goals and expected outcomes for health education based on the theoretical framework
Community Characteristics	• Consider the characteristics of the population(s) • Consider focusing on populations that are in need of screening and/or rarely or never screened • Assess cancer/screening knowledge and screening adherence • Describe unique barriers for screening/rescreening
Setting	• Consider the location of the health fair • Consider the location with the need for data collection systems, education, or screening services (e.g., survey, medical records, onsite screening) • Assess location and potential reach for target audiences
Promotion of the Event	• Consider channels most relevant to the intended audience • Recruit in advance over time and not at the last minute for greatest impact • Use multiple recruitment channels • Employ the assistance of partners or co-sponsors for recruitment (e.g., their constituents, media channels)
Delivery of Services	• Consider what services should be offer at the health fair • Assess the extent to which services are evidence-based strategies to promote cancer screening (e.g., Community Guide such as group/individual education, reminders) • Partner with other organizations to offer services (e.g., education, screening) • Discuss who is the best deliver of education (e.g., provider, lay health worker, etc.) for increased relevance • Consider registration or check in process to enable outreach to participants after the event for reminders, referrals or follow-up
Evaluation	• Determine what process measure should be monitored or assessed (e.g., reach-attendance, implementation, referrals, receipt of education/screening kits, satisfaction/reactions) • Determine what outcomes will be measured (e.g., knowledge, intentions, screening adherence) and follow-up for longer term evaluation • Assess data or tracking systems to be put in place for tracking and evaluation (e.g., surveys, risk appraisals, technology-based data collection)

## Discussion

This process evaluation reported findings from a diverse set of health fairs aiming to increase breast, cervical and colorectal cancer screenings. The health fairs varied by target population, urban/rural focus, education and type of services provided. Many fairs offered education (group or individual) and/or helped reduce structural barriers (i.e., bringing services to people, translated materials, bilingual staff, etc.) as key components. In general, the majority of participants were satisfied with the services provided at the health fair and 80% of participants thought the fairs were important in their decisions to get screened. Most coordinators perceived the health fairs to be successful in reaching underserved populations and thought that providing screening services to people who would not have access without the fair was one of the most important indicators of success. Overall, the health fairs impacted important behaviors that have been associated with increased screening including reading information about cancer screening, speaking to a provider about cancer screening and talking to others about cancer. These health fairs may have activated them to learn and talk more about cancer and preventive screening.

Our data indicated that many of the special events were able to offer cancer screenings from 2 to 68. These events reduced structural barriers to screening [5] and could be considered as ways to reach those who have not been screened or who have difficulty accessing regular sources of care. Over one third of health fair participants were uninsured (37% of the attendees). Many of these individuals were provided with cancer screening ranging from clinical breast exams to Pap testing which may have been difficult for them to receive through regular health care settings. In addition, several of the fairs were strategic in serving specific population (e.g., Latinas, American Indians, LGBTs) who may be rarely or never screened. Health fairs may help to provide education and services targeting vulnerable populations and in venues that can provide strategic reach to for health education and behavior change [2].

In addition to screening, we found that among our seven fairs, staff provided stool test kits for colorectal cancer and/or referral to screening programs. While it is helpful to offer these services, health fairs should assess follow-up to these services such as return of stool kits and test results or completed screenings. Some studies have reported on follow-up of participants about screening results or recommended behavioral changes after health fairs; [18, 19] therefore, it is important to consider post-implementation follow-up tasks to increase adherence to screening.

Community engagement, partnerships, relationship building, and increasing organization or service awareness were additional benefits of health fair that health fair coordinators mentioned. Other health fair evaluations have demonstrated similar benefits for partners and health professionals in training from these events [20]. Research has shown that community members’ level of engagement and perceived connectedness are protective health factors that can decrease risk for developing chronic disease for minority populations [21]. This may be particularly true for health fairs targeting special groups. We found that the health fairs planned for cultural groups were rated higher in satisfaction ratings overall than others and participants enjoyed the cultural aspects (e.g., native foods, activities).

Our process evaluation employed a comprehensive approach by using three data sources, including 6 month follow-up surveys, key informant interviews with fair coordinators, and administrative data from each health fair regarding the attendance, number and type of screening services provided onsite. This approach is more comprehensive than typical health fair evaluations [13, 15] that only collect data on number and type of services provided, participants’ attendance and satisfaction. Our study is one of the first to longitudinally measure screening-related behavior changes of participants after the health fairs and contributed to the body of literature with complementary perspectives from the participants and coordinators. Similar to another health fair evaluation with follow-up assessments [21], our results indicate that health fairs can provide preventive screenings and lead to some behavioral changes influencing screening [22].

Despite benefits, health fairs also have limitations and can be ineffective at changing long term behavior [23]. For example, health fairs that rely solely on education through brochures, group education, or consultation, have been shown to have little or no impact on screening rates [23]. Further, it is unknown whether participants complete the recommended screening as a result of health fairs in the long term. Health fairs have been found to be effective in increasing screening when they offer onsite screening or referrals to screening, a method to reduce barriers to screening [7], as recommended by the Community Guide [5]. Therefore, health fair coordinators should prioritize the recruitment of clinical partners who are able to provide onsite screening and/or provide referrals to screenings or also enroll participants in screening programs or medical homes.

There are several study limitations. The process data only were conducted with a follow-up survey on a proportion of the participants who attended the health fairs and completed the baseline survey (42%), due to constraints of the study design and funds for follow-up. Our sample was also mostly female and middle-aged and may not be representative of all the participants of health fairs for cancer screening. Given that the process evaluation questions were collected in the follow-up survey only, responses may be subject to recall bias 6 months post heath fair. Finally, behavior changes related to being activated about screening among participants reported at follow-up were based on self-report. No metrics have been used to verify these behaviors and they might be subject to social. 

Future research should examine the role that health fairs play in increasing screening-related knowledge and attitudes, increasing preventive health behaviors, and promoting longer-term screening adherence. Process evaluation can provide rich information for learning about program implementation, dose and reactions [24, 25]. These types of evaluations help coordinators learn about successful fair components, refine activities and components for future health fairs and plan for plausible solutions to address potential barriers. Furthermore, more evaluation of different iterations of health fairs could be explored to identify and better understand the most successful activities and components.

## Conclusion

This study provides meaningful data and useful guidance for health promotion practice. Health fairs can be effective in increasing knowledge, changing attitudes or social norms, or modifying other factors associated with cancer screening. They can also support actual screening through onsite services or by facilitating later screening completion through referrals. This mixed methods process evaluation was instrumental in learning more about the reach, participants’ satisfaction and reactions, delivery of services, and lessons learned for health fairs focused on cancer prevention. Continued evaluation research on health fairs and their impacts on screening will contribute to the knowledge base of this common health promotion strategy.

## Abbreviations

CDC: Centers for Disease Control and Prevention
FIT: Fecal immunochemical test
FOBT: Fecal occult blood test
LGBT: Lesbian, gay, bisexual, transgender
SPSS: Statistical Package for the Social Sciences
US: United States

## References

[1] American Cancer Society (2015). Cancer facts & figures 2015.

[2] American Cancer Society (2014). Colorectal Cancer Facts & Figures 2014–2016.

[3] Whitlock EP, Lin JS, Liles E, Beil TL, Fu R (2008). Screening For colorectal cancer: a targeted updated systematic review for the U.S. preventive services task force. Ann Intern Med.

[4] Brown ML, Klabunde CN, Cronin KA, White MC, Richardson LC, McNeel TS (2014). Challenges in meeting healthy people 2020 objectives for cancer-related preventive services, National Health Interview Survey, 2008 and 2010. Prev Chronic Dis.

[5] Sabatino SA, Lawrence B, Elder R, Mercer SL, Wilson KM, DeVinney B, Melillo S, Carvalho M, Taplin S, Bastani R, Rimer BK, Vernon SW, Melvin CL, Taylor V, Fernandez M, Glanz K (2012). Community preventive services task force. Effectiveness of interventions to increase screening for breast, cervical, and colorectal cancers: nine updated systematic reviews for the guide to community preventive services. Am J Prev Med.

[6] Escoffery C, Rodgers KC, Kegler MC, Haardörfer R, Howard DH, Liang S (2014). A systematic review of special events to promote breast, cervical and colorectal cancer screening in the United States. BMC Public Health.

[7] Escoffery C, Rodgers K, Kegler MC, Haardörfer R, Howard D, Roland KB (2014). Key informant interviews with coordinators of special events conducted to increase cancer screening in the United States. Health Educ Res.

[8] Kittleson MJ, Carver VC (1990). The purpose of health fairs as perceived by university-based health educators. Health Values.

[9] Chapman LS (2011). The use of health fairs in health promotion. Am J Health Prom.

[10] Murray K, Liang A, Barnack-Tavlaris J, Navarro AM (2014). The reach and rationale for community health fairs. J Cancer Educ.

[11] Berwick DM (1985). Screening in health fairs: a critical review of benefits, risks, and costs. JAMA.

[12] Dillon DL, Sternas K (1997). Designing a successful health fair to promote individual, family, and community health. J Community Health Nurs.

[13] Fournier AM, Harea C, Ardalan K, Sobin L (1999). Health fairs as a unique teaching methodology. Teach Learn Med.

[14] Flores E, Espinoza P, Jacobellis J, Bakemeier R, Press N (2006). The greater Denver Latino cancer prevention/control network. Cancer.

[15] Elmunzer BJ, T O'Connell M, Prendes S, Saini SD, Sussman DA, Volk ML, Deshpande A. Improving access to colorectal cancer screening through medical philanthropy: feasibility of a flexible sigmoidoscopy health fair for uninsured patients. Am J Gastroenterol, 2011;106(10):1741–1746. 10.1038/ajg.2011.147 21979199

[16] Gosline MB, Schank MJA (2003). University-wide health fair: a health promotion clinical practicum. Nurse Educ.

[17] Corp IBM (2013). IBM SPSS statistics for windows, version 22.0.

[18] Seo DC (2011). Lessons learned from a black and minority health fair's 15-month follow-up counseling. J Natl Med Assoc.

[19] Huang CL (2002). Health promotion and partnerships: collaboration of a community health management center, county health bureau, and university nursing program. J Nurs Res.

[20] Mendez-Luck CA, Bethel JW, Goins RT, Schure MB, McDermott E (2015). Community as a source of health in three racial/ethnic communities in Oregon: a qualitative study. BMC Public Health.

[21] TY W, Kao JY, Hsieh HF, Tang YY, Chen J, Lee J, Oakley D (2010). Effective colorectal cancer education for Asian Americans: a Michigan program. J Cancer Educ.

[22] Bryan JM, Deveraux JM, York ML, Schoh RJ (1991). How effective are health fairs? Quantitative evaluation of a community health fair. Am J Health Prom..

[23] Burron A, Chapma LS (2011). The use of health fairs in health promotion. Am J Health Prom.

[24] Saunders RP, Evans MH, Josh P (2005). Developing a process-evaluation plan for assessing health promotion program implementation: a how-to guide. Health Promot Pract.

[25] Linnan L, Steckler A, Steckler AB, Linnan L, Israel B (2002). Process evaluation. Process evaluation for public health interventions and research.

